# Feature relevance XAI in anomaly detection: Reviewing approaches and challenges

**DOI:** 10.3389/frai.2023.1099521

**Published:** 2023-02-08

**Authors:** Julian Tritscher, Anna Krause, Andreas Hotho

**Affiliations:** Data Science Chair, University of Würzburg, Würzburg, Germany

**Keywords:** explainable artificial intelligence, XAI, feature relevance, anomaly detection, artificial intelligence, machine learning, review

## Abstract

With complexity of artificial intelligence systems increasing continuously in past years, studies to explain these complex systems have grown in popularity. While much work has focused on explaining artificial intelligence systems in popular domains such as classification and regression, explanations in the area of anomaly detection have only recently received increasing attention from researchers. In particular, explaining singular model decisions of a complex anomaly detector by highlighting which inputs were responsible for a decision, commonly referred to as local *post-hoc* feature relevance, has lately been studied by several authors. In this paper, we systematically structure these works based on their access to training data and the anomaly detection model, and provide a detailed overview of their operation in the anomaly detection domain. We demonstrate their performance and highlight their limitations in multiple experimental showcases, discussing current challenges and opportunities for future work in feature relevance XAI for anomaly detection.

## 1. Introduction

Within the last years, artificial intelligence (AI) systems have transformed from simple and interpretable decision systems to complex and highly opaque architectures that are commonly comprised of millions of parameters (Arrieta et al., 2020). With increasing deployment of these highly performing opaque AIs in practice, many application areas have identified a need for explaining the reasoning of complex AI systems. Motivations for explaining these systems range from reducing manual inspection efforts in domains such as medicine (Tjoa and Guan, 2021), to legal requirements for AIs that significantly affect users (Goodman and Flaxman, 2017). As a result, explainable AI (XAI) has become a popular area of research. While the field itself has a longer history with several early applications (Setiono and Leow, 2000; Féraud and Clérot, 2002; Robnik-Šikonja and Kononenko, 2008), a lot of research has been conducted in the last 6 years to provide explanations mainly for common AI tasks such as classification and regression problems (Arrieta et al., 2020). In the area of anomaly detection, research on explainability has taken off more recently, motivated through use in critical security applications such as intrusion and fraud detection (Antwarg et al., 2021), and the desire to decrease manual investigation efforts by domain experts that inspect found anomalies (Sipple and Youssef, 2022).

With the increasing interest on explaining anomaly detection within recent years, first works have started to categorize this emerging research field. While Sejr and Schneider-Kamp (2021) discuss the process of explaining anomaly detection from a user perspective, Nonnenmacher et al. (2022) aggregate anomaly detection XAI work that was specifically designed for tabular data. Panjei et al. (2022) and Yepmo et al. (2022) both provide a general overview of the field of anomaly XAI that categorizes the general types of explanations that may be used to explain anomaly detectors, splitting XAIs by the granularity of their given outputs. Panjei et al. (2022) discuss explanations that return a ranking of found anomalies, XAIs that find causal interactions of outliers, and methods that find relevant features. They focus largely on white box models that find characteristics of outliers in big data. Yepmo et al. (2022) provide an illustrated introduction to four general types of anomaly explanations, e.g., ones that return relevant features or decision rules, and name representative approaches. The authors discuss limitations of the general types of explanations only at a high level, without distinguishing between different approaches. In contrast, we focus on reviewing one specific type of anomaly explanation in-depth. This focused view allows us to construct a fine-grained systematic categorization of different algorithmic approaches and investigate each algorithm in detail. Our review highlights low level limitations of XAI algorithms in anomaly detection that constitute relevant areas for future work.

In this work, we provide an in-depth review of approaches that produce explanations commonly referred to as *local post-hoc feature relevance XAIs* (Arrieta et al., 2020) in the field of anomaly detection. While a variety of XAIs exist that yield different types of explanations as output, *feature relevance XAIs* explain the decision process of anomaly detection models through highlighting relevant input features, providing as output a relevance score for each input feature. They constitute the currently most used type of explanation in anomaly detection (Yepmo et al., 2022). Applying feature relevance XAIs in a *local* fashion, i.e., per data point, results in highlighting relevant input features that lead an anomaly detection model to identifying a singular data point as an anomaly, in contrast to XAIs that provide a global explanation of general model behavior. This provides additional information regarding a singular found anomaly to manual investigators and reduces their inspection efforts. Further, in contrast to *ante-hoc* approaches that describe inherently explainable anomaly detectors such as simple linear models, *post-hoc* XAIs describe dedicated XAI approaches that are applied to already fully trained anomaly detectors, allowing the use of highly complex and well performing model architectures without constraining their complexity during model training. The resulting sub-field of local *post-hoc* feature relevance XAI, which we will refer to in abbreviated form as *feature relevance XAI* in the remaining paper, has recently received increasing attention within the domain of anomaly detection. We systematically review approaches from this sub-field that have been applied to anomaly detection in the remaining paper.

**Figure 1:**
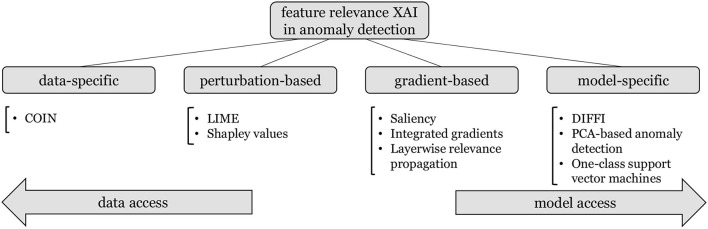
Overview of the reviewed feature relevance approaches in anomaly detection, structured by their use of information from data and from the underlying anomaly detection model.

We provide a structured characterization of the reviewed approaches in Figure 1, where we group approaches based on their reliance on training data on one hand, and on the anomaly detection architecture on the other hand. This categorization leads from completely model-agnostic XAI approaches that utilize additional assumptions and information obtained through training data, to model-specific XAIs that heavily rely on the model structure to obtain feature relevance explanations. Additionally, we identify two groups of hybrid approaches that access both the model and information regarding the underlying data. While perturbation-based approaches restrict their model access purely to allowing inference on data that may be augmented according to data assumptions, gradient-based approaches require access to the first order derivatives of differentiable anomaly detection models. We provide an in-depth introduction to these groups of approaches, and demonstrate their limitations and challenges in multiple showcases to outline future research directions of feature relevance XAI in anomaly detection.^1^

The remainder of the paper is structured as follows: Section 2 formally introduces the tasks of anomaly detection and feature relevance explanations, as well as the data, model architectures, and performance metrics we use in our showcases. Section 3 covers data-specific approaches that possess no access to the anomaly detection model, instead generating their explanations through training data. Section 4 introduces perturbation-based approaches that generate explanations through repeatedly querying the anomaly detection model with altered, so called *perturbed* data points. Section 5 includes gradient-based approaches that require differentiability of the anomaly detection model and utilize gradients that contain knowledge of the inner model structure to obtain explanations. Section 6 presents model-specific approaches that are developed for specific model architectures and take full advantage of the model structure to generate their explanations. In Section 7 we conclude by discussing the overarching limitations of feature relevance explanations in anomaly detection and highlighting future research areas within the domain.

## 2. Methodology

Before we review existing feature relevance XAI approaches, we briefly define the tasks of anomaly detection and feature relevance XAI, as well as give a brief overview of the data, anomaly detection models, and XAI evaluation metrics we use to showcase XAI approaches and their limitations throughout this study.

### 2.1. Anomaly detection

Anomaly detection, as laid out by Chandola et al. (2009), describes the task of identifying anomalous behavior in data that contains well-defined normal behavior. For data points $x\in X\subseteq {\mathbb{R}}^{d}$ of dimensionality *d*, anomaly detection is the identification of anomalous data points through a model *m*(*x*), where *m*(*x*) may be either modeled as a binary classification, a probabilistic estimation of anomalies, or as a regression task that assigns each point an anomaly score. In this work, we view anomaly detection as regression task m(x):X→[0,inf] with lower scores representing normal data and higher scores for anomalies.

While anomaly detection at a high level is only a subset of classification or regression, the unique challenges in anomaly detection arise from specific data characteristics: only the normal behavior in anomaly detection is well-defined and normal data is typically readily available, but anomalies may vary greatly in behavior with only a small number of anomalies that are known during training. As a result, proposed approaches typically focus on the well-defined normal data to be able to identify potentially unseen types of anomalous behavior, e.g., through encircling observed normal behavior in one-class support vector machines (Schölkopf et al., 2001), assessing the density around data points in kernel density estimation (Terrell and Scott, 1992), or learning a reconstruction of the normal behavior with autoencoder neural networks (Goodfellow et al., 2016).

### 2.2. Feature relevance explanations

Local *post-hoc* feature relevance explanations explain the model prediction *m*(*x*) for a specific input *x* through assigning a score to each input feature, creating an explanation *f*(*x, m*) ∈ ℝ^*d*^ that reflects how much each input feature influenced the final prediction according to model *m*. In the domain of anomaly detection, feature relevance explanations are commonly applied to anomalous data points, and focus on highlighting the relevant features that lead the anomaly detection model to identify the data point as an anomaly (Yepmo et al., 2022).

### 2.3. Data

While there is no shortage of datasets for anomaly detection, most of these do not include ground truth explanations for anomalies. Since this ground truth enables an otherwise challenging direct comparison and quantitative judgment of explanations generated by XAI approaches, we select two datasets for the showcases conducted in this review that offer these ground truth explanations: MVTec (Bergmann et al., 2019) and ERP (Tritscher et al., 2022a).

MVTec (Bergmann et al., 2019) is an anomaly detection dataset for industrial visual fault detection. The dataset contains 15 texture and object classes with the training set for each category containing only normal images, e.g., without defects, and the test set containing images with defects and without defects. The defects are annotated with manually created ground truth pixel maps, with binary indications of pixels that are part of the defect. The dataset has been previously used to evaluate feature relevance XAI approaches by Ravi et al. (2021), although their evaluations are limited to qualitative inspections of results. To instead generate quantitative results of XAI performance, we use the ground truth anomaly segmentation maps as ground truths for explanations. For our showcases, we focus on the *grid* class from the dataset that contains 264 high resolution images of normal wire mesh for training and 57 images with different faults and ground truth for testing. We choose this class as it has the highest detection accuracy of the used anomaly detection model. This limits the influence of poor model performance on the quality of the obtained explanations, which we motivate further in Section 2.4.

ERP (Tritscher et al., 2022a) is a synthetic enterprise resource planning (ERP) dataset generated by using a serious game within a real ERP system (Léger et al., 2007). The data includes financial documents from a simulated production company, where different financial fraud scenarios have been committed within the simulation. Additionally, the provided fraudulent data points come with ground truth features that are indicative of the fraud case according to auditing experts, which we utilize as ground truth explanations. For analysis in this work, we rely on the joint machine-learning ready data provided by Tritscher et al. (2022a) that focuses on the financial accounting data. We utilize their run *normal 2* that contains 32, 337 data points of purely normal operation for training the anomaly detector and evaluating explanations on the 86 different fraud cases contained in their run *fraud 3*. We choose these runs following the experimental setup of Tritscher et al. (2022b), again using *fraud 3* as the dataset with highest performance of the used anomaly detection model and the corresponding normal behavior of *normal 2*.

### 2.4. Models

To showcase XAI algorithms on the introduced data, we select an anomaly detection model with high detection performance through common metrics such as AUC-PR and AUC-ROC scores from literature for each dataset. We specifically require high performance from our anomaly detectors to not obscure the quantitative XAI evaluation. With poorly performing models a miss match of ground truth and explanation may be caused by the model, and not just the XAI approach, preventing the result from reflecting the XAI performance.

For the MVTec image dataset, Kauffmann et al. (2020b) train kernel density estimation (Rosenblatt, 1956), deep support vector data description (Ruff et al., 2018), and autoencoder neural networks (Goodfellow et al., 2016) on MVTec data. While their models show high anomaly detection performance, further analyzes by the authors reveal that their models and model ensembles use spurious correlations in the data, which may skew a quantitative XAI evaluation. Wang et al. (2021) propose a student-teacher neural network that is designed for segmenting anomalous regions within the MVTec images. The network incorporates a teacher network that consists of three pre-trained feature extraction layers from the popular ResNet-18 architecture (He et al., 2016) and a randomly initialized student that possesses the same network architecture as the teacher and is trained to mimick the pre-trained teacher on normal training data. While the resulting student-teacher architecture directly outputs image segmentation maps with highlighted anomalous regions, it can be adapted to image-level anomaly detection through adding a mean pooling step to the final output. This creates a well-performing image-level anomaly-detector that is capable of finding anomalies within the MVTec data both on an image- and a pixel-level and can be used as a test-bed for the investigated XAIs.

For the ERP dataset, Tritscher et al. (2022b) conduct a hyperparameter study of multiple anomaly detectors on the data, finding architectures that yield good results on the dataset. For our showcases, we select their second best performing model, the autoencoder neural network (Goodfellow et al., 2016) architecture, with their found hyperparameters as they show that their best performing one-class support vector machine (Schölkopf et al., 2001) exibits an erratic decision process that may influence a quantitative XAI evaluation and autoencoder networks are commonly studied in the domain of explainable anomaly detection (Antwarg et al., 2021; Ravi et al., 2021; Müller et al., 2022).

### 2.5. Evaluation metrics

To showcase the performance of different feature relevance XAI approaches, we utilize the binary ground truth explanations contained in the datasets that denote for each input feature whether the feature was indicative of the underlying anomaly (1) or part of normal behavior (0). To generate quantitative results with this type of ground truth explanation, a performance metric for comparing ground truth with generated explanations is required.

Hägele et al. (2020) use the well-known area under the receiver operating characteristic (ROC) as metric for their feature relevance evaluation on medical image data. As ROC scores are calculated using the true positive rate over increasing threshold values, early true positives are more impactful to the resulting area under the curve. When applied to feature relevance, this corresponds to a stronger focus on finding truly relevant features within the top scoring features of a given explanation. This is an intuitive metric, as anomaly detectors do not need to identify all anomalous features within an anomaly, but may sufficiently detect the anomaly by focusing heavily on few features that are indicative of the anomalous behavior.

To complement the ROC score, we also report cosine similarity (COS) as used for feature relevance evaluation by Kauffmann et al. (2020b), which reflects the similarity of the found feature relevance explanations to the entire ground truth. Intuitively, this corresponds to how well an obtained explanation finds all truly anomalous features. This metric also holds interesting properties in the case of non-binary ground truths, since COS respects the magnitudes of the ground truth feature relevance.

Both metrics can be calculated for each data point individually, and can then be aggregated across multiple anomalous data points. In this work, we therefore report mean and standard deviation of the resulting metrics across all anomalies.

## 3. Data-specific explanations

Data-specific explanations identify relevant feature values of anomalies entirely through training data without any access to the anomaly detection model. The anomalies themselves are found by an anomaly detection model, effectively making data-related explanations *post-hoc* XAIs. However, these approaches act independently of the anomaly detection model and identify relevant features in given anomalies entirely through their own assumptions.

### 3.1. Contextual outlier interpretation

Contextual Outlier INterpretation (COIN) (Liu et al., 2018), to our knowledge currently the only data-specific *post-hoc* feature relevance XAI approach, explains an anomalous data point *x* found by an anomaly detection model *m* by determining how much it's input features are responsible for separating *x* from training data $X_{train}$. As a first step, COIN extracts context data points C from the normal data within $X_{train}$ that are close to *x* in feature space through nearest neighbors such that $nn\left(x,X_{train}\right)=C$. Since several distinct types of normal behavior might exist in the data, COIN then uses clustering $cl\left(C,c\right)=C_{c}$ to separate the context data points C into individual groups *c* with similar behavior. For each of these groups, a decision boundary separating $C_{c}$ from the anomaly *x* is learned via a linear support vector machine *s* (Boser et al., 1992) with loss $L_{s}\left(x,C_{c}\right)$ and an L1 regularization term Ω(*s*) through


(1) $$\begin{array}{l} S_{c}\left(x\right)=\underset{s}{\arg\min}L_{s}\left(x,C_{c}\right)+\Omega \left(s\right). \end{array}$$

Letting $w_{c}\in {\mathbb{R}}^{d}$ denote the weights of the resulting linear support vector machine *S*_*c*_ for context group *c*, the relevance of individual feature values within *x* are then obtained through the weights of the SVM through


(2) $$\begin{array}{l} f_{c}\left(x_{i}\right)=abs\left(w_{c,i}\right)/\gamma_{c,i}, \end{array}$$

Where γ_*c, i*_ denotes the average distance between data points in $C_{c}$ for the *i*th feature. To obtain the final feature relevance scores of anomaly *x*, the feature relevance scores of individual context groups are averaged. This results in the following process for feature relevance explanations:


(3) $$\begin{array}{l} f_{COIN}\left(x_{i},X_{train}\right)=\left(1/|nn\left(x,X_{train}\right)|\right)\underset{c}{\sum }|cl\left(nn\left(x,X_{train}\right),c\right)|\cdot f_{c}\left(x_{i}\right). \end{array}$$

### 3.2. Limitations

Data-specific feature relevance XAIs explain found anomalies purely from the data domain, and are therefore applicable without any access to the anomaly detection model. Due to this complete separation of anomaly detection model and explanation approach, the XAI needs to build its feature relevance explanations purely relying on given data. As observed in the introduced COIN framework, this requires additional assumptions regarding the data in multiple steps during the explanation process. Since COIN relies both on a nearest neighbors algorithm to identify the local context data points around a given anomaly, and on clustering to separate multiple distinct types of normal behavior in the data, this requires the definition of a meaningful distance function within the data. Obtaining reasonable assumptions regarding the distance metric for a given dataset is a non-trivial task, effectively requiring the construction of an additional, well-performing, distance-based anomaly detection system to obtain high quality explanations for a given dataset. As a result, if such a well-performing distance-based anomaly detection system is not available, e.g., in domains where distance-based anomaly detectors perform poorly in general, an application of the COIN framework may yield poor results due to it's internal reliance on the construction of an additional anomaly detector.

## 4. Perturbation-based explanations

In contrast to data-driven approaches that only access the final decision of an anomaly detection model *m*(*x*) for a given anomalous data point *x*, perturbation approaches allow free access of the model decision function *m* on arbitrary data points. While this does not provide direct knowledge on the structure of the anomaly detection model, effectively treating *m* as a black box, it provides an opportunity to probe the model behavior. Perturbation approaches use the access to the anomaly detection function *m* by repeatedly constructing synthetic data points *x*′ through altering the given anomalous data point *x*, and probing the anomaly detection model's reaction to the alterations by applying the model to the synthetic data points through *m*(*x*′).

To obtain relevance scores for individual features, this probing procedure is used to remove features and feature combinations from the anomaly *x* and measure the model's reaction to the presence and absence of features. Perturbation approaches alter an anomalous data point *x* = [*x*_1_, *x*_2_, …, *x*_*d*_] of dimensionality *d* by determining a set of features *K* ⊆ {1, 2, …, *d*} to keep, and subsequently deleting, i.e., perturbing, the *K*^*C*^ remaining features not in *K* from data point *x*, where *K*^*C*^ denotes the complement of *K* (i.e., *K*^*C*^ = {1, 2, …, *d*}\*K*). This perturbation procedure is used by several XAIs repeatedly on a single data point *x* to gather information on the behavior of the machine learning model when specific feature values within *x* are removed, allowing them to identify single features and feature groups that determine the model output. Since a large amount of machine learning models are not capable of handling missing values, the construction of perturbed data points is commonly achieved not through deletion but through replacing the values in *K*^*C*^ with additional reference data *r* ∈ ℝ^*d*^ through $h\left(x,r,K\right)=\left[x_{K},r_{K^{C}}\right]$.

### 4.1. Local interpretable model-agnostic explanations

Local Interpretable Model-agnostic Explanations (LIME) (Ribeiro et al., 2016) generates explanations for model decisions on single data points *x* through the perturbation procedure. LIME generates a synthetic dataset $X^{\prime }$ around anomaly *x* through $s:{\mathbb{R}}^{d},{\mathbb{R}}^{d}\to X^{\prime }$ by perturbing *x* with reference data *r* and sampling the features to perturb from a uniform distribution such that


(4) $$\begin{array}{l} s\left(x,r\right)=X^{\prime }\sim U\left(\left\{h\left(x,r,K\right),K\subseteq \left\{1,2,\ldots ,d\right\}\right\}\right). \end{array}$$

These synthetic data points are then weighted through a proximity measure π_*x*_ that indicates the proximity of the synthetic points to the original data point *x* to explain. Using this synthetic data, an explanation is then obtained through the parameters of a linear and therefore interpretable model with linear coefficients *w* ∈ ℝ^*d*^ and bias *b* ∈ ℝ, that is trained to mimick the original model *m* on the synthetic data points $X^{\prime }$ in the proximity π_*x*_ through a loss function $L\left(m,w,b,X^{\prime },\pi_{x}\right)$. This linear model is regularized through a complexity measure Ω(*w*), which enforces simple and readily interpretable linear coefficients *w*. As a result, LIME generates explanations for a data point *x* by linearly approximating the original model *m* in the local proximity π_*x*_ through


(5) $$\begin{array}{l} \left(W\left(x,m,r\right),B\left(x,m,r\right)\right)=\underset{\left(w,b\right)}{\arg\min}L\left(m,w,b,s\left(x,r\right),\pi_{x}\right)+\Omega \left(w\right). \end{array}$$

This results in a local linear model with one linear coefficient for each input feature. As a result, the linear coefficients show the relevance of each feature in the local vicinity of $X^{\prime }$ and can be taken directly as feature relevance explanations through


(6) $$\begin{array}{l} f_{LIME}\left(x,m,r\right)=W\left(x,m,r\right). \end{array}$$

Ravi et al. (2021) directly apply LIME on the anomaly detection MVTec dataset with a brief qualitative demonstration of results. Further, Zhang et al. (2019) apply LIME on multiple anomaly detection datasets from the security domain that focus on intrusion and malware detection. While LIME yields both positive and negative contributions to the model output, Zhang et al. (2019) only retain contribution signals that cause an increased anomaly score. They also introduce an additional, optional loss term based on KL divergence that allows for determining the desired distribution of output explanation scores.

### 4.2. Shapley value explanations

The Shapley value (Shapley, 1997), a well-known result from cooperative game theory, describes a unique solution to fairly distributing cooperatively achieved gain among *n* cooperating players by measuring the achieved gain of partial coalitions. The solution provided by Shapley uniquely satisfies desirable fairness properties such as permutation in-variance of coalitions and zero gain for players not included in the coalition, among others. The Shapley value ϕ_*i*_ for a single player *i* represents the gain generated by player *i* and can be computed through iteratively measuring the gain of all coalitions without player *i* in comparison to the same coalition with player *i* included, giving:


(7) $$\begin{array}{l} \phi_{i}=\underset{S\subseteq N\backslash \left\{i\right\}}{\sum }\frac{|S|!\left(n-|S|-1\right)!}{n!}\left(v\left(S\cup \left\{i\right\}\right)-v\left(S\right)\right) \end{array}$$

For the set of all players *N* = {1, 2, …, *n*} and a function *v*(*S*) to compute the gain of a coalition *S*.

Applying Shapley values to the domain of feature relevance explanations, as done by Lundberg and Lee (2017), is achieved by viewing the features of *x* as players, building coalitions through perturbations, i.e., through keeping and replacing features, and computing the gain as the outcome of applying the model on the synthetic data point from the coalition, giving


(8) $$\begin{array}{l} f_{Shapley}(x_{i},m,r)=\sum_{{\;}_{K\subseteq N\backslash \{i\}}}\frac{|K|!(d-|K|-1)!}{d!}(m(h(x,r,K\cup \{i\}))\\ \;-m(h(x,r,K)). \end{array}$$

Since computing the true Shapley value as feature relevance is prohibitively resource-intensive for reasonably sized numbers of features *d*, multiple approaches exist for estimating Shapley values. As the predominant work in XAI, SHapley Additive exPlanations (SHAP) (Lundberg and Lee, 2017) shows that proposing slight alterations to existing XAI approaches can yield approximate Shapley value explanations. For their approach “kernel-SHAP,” the authors adapt the perturbation framework of LIME, showing that LIME is capable of recovering an approximation of Shapley values using the following choices of proximity kernel π_*x*_ and regularization term Ω(*g*) when fitting LIME's linear approximation model in Equation (5):


(9) $$\begin{array}{l} \pi_{x}=\frac{d-1}{\left(\begin{array}{c} d\\ K \end{array}\right)\;\cdot K\;\cdot (d-K)},\;\Omega (g)=0 \end{array}$$

For datasets with high dimensionality *d* and a known hierarchy between dimensions (e.g., local dependencies in images), "partition-SHAP" extends this approach to groups of features through the game-theoretic extensions to Owen values (Owen, 1977) and achieves faster run times as a result.

Shapley value explanations are some of the most used approaches in anomaly detection, with multiple applications on reconstruction-based anomaly detectors such as autoencoder neural networks (Antwarg et al., 2021; Ravi et al., 2021; Müller et al., 2022; Tritscher et al., 2022b). While Ravi et al. (2021) and Tritscher et al. (2022b) apply Shapley value estimation directly on the final anomaly score of the reconstruction-based anomaly detection model, Antwarg et al. (2021) first identify the features with highest reconstruction errors and apply kernel-SHAP directly on the most deviating features. Müller et al. (2022) further extend this approach to categorical one-hot encoded data by averaging over groups of one-hot encoded features.

### 4.3. Showcase and limitations

#### 4.3.1. Showcase of perturbation approaches

To be able to discuss the application of perturbation approaches to anomaly detection and showcase the resulting limitations in detail, we first demonstrate the performance of the two previously introduced approaches LIME and SHAP using their default parameter settings on the datasets MVTec and ERP described in Section 2.3. While we use kernel-SHAP for all applications of SHAP on ERP data, we use the authors' partition-SHAP implementation for the large image dataset of MVTec to maintain computational feasibility. We compute feature relevance explanations on all anomalies in the respective test datasets and compare the resulting explanations to ground truth using the ROC score and cosine similarity as discussed in Section 2.5. Additionally, to ease interpretablility of results, we introduce two random baselines that include explanations sampled from random uniform noise, as well as a multiplication of random uniform noise with the anomalous input itself (noise × input). Table 1 shows that both LIME and SHAP are capable of highlighting relevant features on ERP data, demonstrating considerably higher scores then random noise. On the image data of MVTec, however, both approaches perform poorly on all metrics with only small improvements over the random baselines.

**Table 1:** Mean and standard deviation of perturbation XAI performance comparing to ground truth explanations over all anomalies for ERP and MVTec data, respectively.

**XAI**	**ERP**		**MVTec**	
	**ROC**	**COS**	**ROC**	**COS**
Noise	22.7 (7.0)	–28.7 (5.3)	50.2 (1.8)	6.3 (2.7)
Noise × input	52.3 (13.0)	–27.2 (8.2)	32.3 (10.7)	–4.5 (5.5)
LIME	**75.7 (3.9)**	28.6 (8.3)	56.6 (9.2)	**10.9 (13.7)**
SHAP	74.4 (17.1)	**32.3 (25.1)**	**64.5 (19.9)**	–5.3 (2.8)

Best results highlighted in bold.

#### 4.3.2. Limitation: Choice of reference values *r*

**Figure 2:**
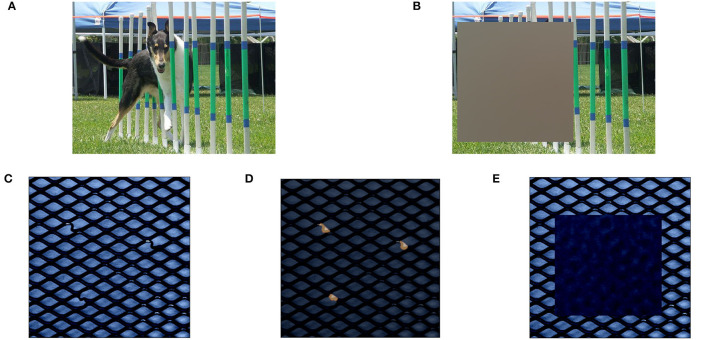
Demonstration of perturbation with mean reference *r* in classification **(A, B)** and anomaly detection **(C, E)**. In classification, mean reference is capable of completely removing the class signal “dog” in image **(B)**. In anomaly detection, replacing all areas that contain anomalies [highlighted in image **(D)**] with mean reference *r* introduces new anomalous signals in the resulting data point in image **(E)**.

One key aspect of perturbation-based explanation approaches is the choice of reference data *r* for removing signal and representing missing information, which is a non-trivial question that is still unsolved in current research (Ancona et al., 2019). Common references that stem from well researched tasks such as image classification include replacing feature values with zero values or averages obtained from training data (used by LIME and SHAP as default in Table 1). We demonstrate this on an image of a dog in Figure 2A, taken from the well-known ImageNet classification dataset (Russakovsky et al., 2015). When classifying a dog within the image, perturbing features through mean values from data as reference *r* (here calculated from the ImageNet validation split as demonstration), intuitively removes any signal present in the replaced features that might be indicative of the dog (see Figure 2B). As a result, mean values are capable of removing the relevant signal on perturbed input features in this setting.

Within the domain of anomaly detection, however, these fixed reference values might introduce unwanted signals into the data. We demonstrate this on an anomalous data point from the MVTec test dataset that contains a bent wire anomaly in an otherwise normal wire mesh (Figure 2C). The anomalous inputs according to the ground truth explanations are highlighted in Figure 2D. Replacing a region that covers all anomalous inputs with mean values from the MVTec training data may still yield an anomalous data point *x*′ that does not represent the well-defined normal behavior of a wire mesh (see Figure 2E). Even though all inputs that contain anomalous entries have been replaced from the initial anomaly, the resulting image may still be declared as anomaly by the model and therefore prevent XAI approaches from finding the relevant anomalous inputs.

#### 4.3.3. Finding optimal reference values *r* in anomaly detection

**Figure 3:**
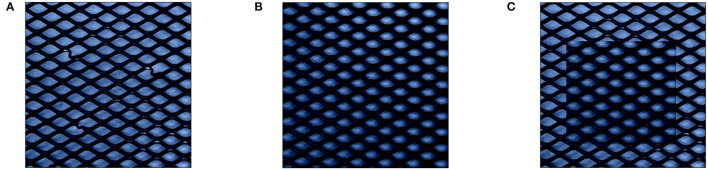
Demonstration of perturbation with nearest neighbor in normal train data as reference *r*: [**(A)** Anomalous MVTec image *x*, **(B)** nearest neighbor as *r*, and **(C)** *x*′ with nearest neighbor as *r*.] While *r* is visually closer to the anomaly *x* than the mean of training data from Figure 2E, the perturbed point *x*′ still shows highly anomalous characteristics on the replacement borders.

To alleviate this issue, reference values *r* have in the past been chosen in the context of the data point *x*, e.g., through finding nearest neighbors to *x* within normal training data that is both similar to *x* and lies within the normal data manifold (Takeishi and Kawahara, 2020). While this can indeed produce normal data points after perturbation for some groups of retained feature values *K* (e.g., when replacing all values within *x*), for some values of *K* the combination of anomalous data point *x* and it's nearest neighbor might still introduce further unwanted anomalies as visualized in Figure 3.

To achieve better perturbation-based explanations, Takeishi and Kawahara (2020) propose to find *r* dynamically dependent on the data point *x* and features to keep *K*. To additionally ensure that the perturbed features make the resulting data point more normal, Takeishi and Kawahara (2020) generate the synthetic data point $x_{opt}^{\prime }$ by minimizing the model output in the local neighborhood $N_{x}$ of the original data point while constraining the features in *K* to their original values in *x*, giving


(10) $$\begin{array}{c} {x^{\prime }}_{opt}=\underset{\hat{x}\in N_{x}}{\arg\;\min}\;m(\hat{x})\;s.t.\;{\hat{x}}_{i}=x_{i},\forall i\in K. \end{array}$$

Takeishi and Kawahara (2020) further relax this generation procedure by searching for a local minimum of Equation (10) instead through


(11) $$\begin{array}{c} x_{lopt}^{\prime }=\arg\min m\left(\hat{x}\right)+\gamma \cdot dist\left(x,\hat{x}\right) \end{array}$$

Using a distance function *dist*:ℝ^*d*^ × ℝ^*d*^ → ℝ, which may be minimized through constrained optimization with the constraints ${\hat{x}}_{i}=x_{i},\forall i\in K$. To further reduce the computational overhead required for synthetic data generation on data points with reasonably low dimensionality (*d* < 500), they additionally propose to only carry out optimizations using Equation (11) while keeping single features individually (i.e., setting |*K*| = 1) and constructing synthetic data points through


(12) $$\begin{array}{l} x_{i}^{\prime }=\{\begin{array}{ll} x_{i} & \text{if}i\in K\\ \frac{1}{|K|+1}\cdot \left(x_{lopt}^{\prime }\left(\emptyset \right)+\sum_{i\in K}x_{lopt}^{\prime }\left(\left\{i\right\}\right)\right) & \text{else.} \end{array} \end{array}$$

To demonstrate the effect of these different choices of reference values, we conduct an additional showcase using SHAP with different reference values *r*. Next to the mean of training data (mean), we demonstrate SHAP's performance when using the zero vector as reference (zeros), which is another common choice in classification and regression settings. We also evaluate nearest neighbors of the normal training data (NN) as choice of reference, and integrate the approach of Takeishi and Kawahara (2020) into SHAP (lopt). For the lower dimensional ERP dataset we integrate the approach of Equation (12) into kernel-SHAP. For the larger dimensional MVTec dataset we integrate Equation (11) into partition-SHAP. Observing the results in Table 2, we notice that while zero values yield good explanations on ERP data the optimization procedure of Takeishi and Kawahara (2020) is capable of further improving results. For the image dataset MVTec, however, only minor increases on some performance metrics are observed, with the overall explanations still very poorly correlating with the ground truth.

**Table 2:** Mean and standard deviation of SHAP performance for ERP and MVTec data when using mean of training data (mean), zero vector (zeros), nearest neighbor in training data (NN), and optimized data points (lopt) as reference *r*.

***r***	**ERP**		**MVTec**	
	**ROC**	**COS**	**ROC**	**COS**
Mean	74.4 (17.1)	32.3 (25.1)	64.5 (19.9)	–5.3 (2.8)
Zeros	82.1 (14.2)	58.2 (16.3)	**67.8 (19.9)**	–2.8 (3.4)
NN	56.0 (15.1)	16.8 (38.0)	66.8 (15.5)	–3.3 (3.5)
lopt	**88.6 (11.2)**	**66.1 (20.5)**	57.7 (21.4)	**4.4 (8.3)**

Best results highlighted in bold.

**Figure 4:**
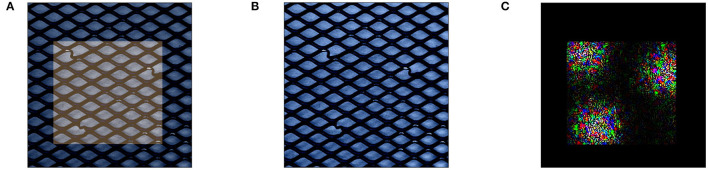
Perturbation with Equation (11) on MVTec: when perturbing *x* while keeping the dark area *K* shown in **(A)**, Equation (11) generates data point $x_{lopt}^{\prime }$ that is visually indistinguishable from *x* **(B)**. We visualize the amplified change in pixel values in **(C)**.

Investigating the generated data points $x_{lopt}^{\prime }$ for the MVTec data in detail reveals that this approach produces many adversarial examples, i.e., examples that appear normal to the anomaly detection model, but do not truly conform to the characteristics of normal behavior. We demonstrate this behavior on our previously used anomaly *x* in Figure 4. Here, optimization yields a data point $x_{lopt}^{\prime }$ that is visually indistinguishable from *x*, with actual differences between the points enlarged in Figure 4C. This adversarial behavior indicates that the method relies on areas where the decision boundary of the underlying anomaly detection model *m* is not capable of generalizing and falsely associates data points with anomalous characteristics within the normal data.

As the generation of adversarial samples might skew the resulting explanations, future research might gain improvements over the work of Takeishi and Kawahara (2020) by specifically tuning the optimization process to find plausible inputs, which is a common technique used within the research area of counterfactual explanations (Guidotti, 2022). Additionally, the procedure of Takeishi and Kawahara (2020) introduces a large computational overhead for perturbation approaches that take thousands of sampled *x*′ values for each data point *x* to explain. Further improving the performance aspects of this procedure is therefore another promising area of research.

## 5. Gradient-based explanations

In contrast to model-agnostic XAI approaches that base their explanations entirely on the input *x* and output of the investigated model *m*(*x*), gradient-based approaches leverage the gradient of the model output with respect to the input $\frac{\partial m\left(x\right)}{\partial x}$ as additional information, therefore requiring investigated models to be differentiable with regards to their input and assuming that the model parameters are available during inference.

### 5.1. Saliency

Simonyan et al. (2014) established the use of the gradient of the output with respect to the input as a way to interpret backpropagation-based anomaly detectors. For their feature relevance explanations on image classification, which they refer to as saliency maps, they take the absolute gradient of the output with respect to the input, using the maximum gradient value for each pixel over all color channels in the case of rgb images:


(13) $$\begin{array}{c} f_{Saliency}\left(x,m\right)=|\frac{\partial m\left(x\right)}{\partial x}| \end{array}$$

Beyond the utilization of the raw gradient, many applications also include a multiplication of the signed gradient values with the original input to achieve a less noisy feature relevance output (Shrikumar et al., 2016), leading to an approach commonly referred to as gradient × input:


(14) $$\begin{array}{c} f_{gradient\times input}\left(x,m\right)=\frac{\partial m\left(x\right)}{\partial x}\cdot x \end{array}$$

Nguyen et al. (2019) employ Saliency to obtain gradient-based feature relevance explanations for variational autoencoder networks on anomaly detection in NetFlow data, and further cluster the obtained feature relevance explanations to identify characteristics of anomalies.

### 5.2. Integrated gradients

Sundararajan et al. (2017) note that Saliency approaches break a desirable sensitivity property that explanation approaches ought to satisfy: when only a single feature is changed within a data point, and this change alters the model's prediction, the feature should obtain a non-zero contribution. Since Saliency may violate this property in areas where the gradients are zero (e.g., around saturated activation functions), Sundararajan et al. (2017) propose a path-based approach. For a given data point *x*, they propose to use a reference data point *r* and define a smooth function giving interpolated data points on the straight-line path between *x* and *r* as γ(*x, r*, α):ℝ^*d*^, ℝ^*d*^, [0, 1] → ℝ^*d*^. Gradients are then calculated for these synthetic data points, and an overall feature relevance explanation accumulated through a path integral


(15) $$\begin{array}{c} f_{IG}\left(x,m,r\right)=\int_{\alpha =0}^{1}\frac{\partial m\left(\gamma \left(x,r,\alpha \right)\right)}{\partial \gamma_{i}\left(x,r,\alpha \right)}\frac{\partial \gamma_{i}\left(x,r,\alpha \right)}{\partial \alpha }d\alpha , \end{array}$$

with $\frac{\partial m\left(x\right)}{x_{i}}$ as the gradient of *m* at *x* along dimension *i*. The resulting approach, called Integrated Gradients (IG), yields feature relevance values that sum to the difference of the model output at the data point to be explained and the output at the reference point.

Sipple (2020) apply IG on anomaly detectors trained through negative sampling by choosing the nearest neighbors of data points in Euclidean space as reference *r*. Sipple and Youssef (2022) motivate the use of IG in anomaly detection from a human perspective and apply IG to real world data while sampling reference points *r* from clustered normal data.

### 5.3. Layerwise relevance propagation

Instead of utilizing the gradient directly for feature relevance attribution, Layerwise Relevance Propagation (LRP) (Bach et al., 2015) utilizes deep Taylor expansion (Montavon et al., 2017) to build feature relevance explanations within neural networks.

Consider a neural network that consists of *L* subsequent layers with $u_{i}^{l}$ being the *i*th intermediate neuron in layer *l* ∈ {1, 2, …, *L*−1}, and where *u*^1^ = *x* denotes the input layer and *u*^*L*^ denotes the output layer. LRP then computes a relevance value $R_{i}^{l}$ for each neuron $u_{i}^{l}$ within the network. To obtain the relevance values for the input layer that correspond to feature relevance explanations $f_{LRP}\left(x,m\right)=R^{1}$, LRP first assigns the relevance of the last network layer to the final model output (*R*^*L*^ = *u*^*L*^ = *m*(*x*)). Then, the entire relevance is propagated to the previous layer recursively while maintaining the same total relevance in each layer ($\underset{i}{\sum }R_{i}^{l}=\underset{j}{\sum }R_{j}^{l+1}$ for all *i* neurons in layer *l* and all *j* neurons in layer *l*+1), called the conservation property of LRP. The actual propagation of relevance to a neuron *i* of the previous layer is realized through a Taylor expansion around a manually chosen root point ${ũ}_{i}^{\left(j\right)}$ with


(16) $$\begin{array}{c} R_{i}^{l}=\underset{j}{\sum }\frac{\partial R_{j}^{l+1}}{\partial u_{i}^{l}}{|}_{{ũ}_{i}^{\left(j\right)}}\cdot \left(u_{i}-{ũ}_{i}^{\left(j\right)}\right). \end{array}$$

While it has been shown that under specific parameter choices LRP is equivalent to the gradient × input approach in Equation (14) (Shrikumar et al., 2016), advantages of this approach are the possibility to manually choose the order of Taylor expansion for each layer, which allows the approach to go beyond the first order approximations of gradients when needed. Additionally, the root point ũ also needs to be chosen manually for each layer, such that the conservation property of LRP is retained.

Amarasinghe et al. (2018) apply LRP in its standard setting on the task of detecting denial of service attacks, but model the task as direct classification using feed forward neural networks instead of anomaly detection architectures. As a direct application on anomaly detection architectures, Ravi et al. (2021) use a standard variant of LRP that is equivalent to gradient × input on autoencoder neural networks trained on the MVTec dataset. To appropriately adjust LRP to the task of anomaly detection, Kauffmann et al. (2020b) propose specific propagation rules for common neural network layers in anomaly detection, and introduce a unifying framework that transfers existing anomaly detectors into neural network representations that use layers for which LRP rules are defined. Through this transfer procedure, they show that LRP is applicable to a wide range of anomaly detectors.

### 5.4. Showcase and limitations

#### 5.4.1. Showcase of gradient-based approaches

To discuss the limitations of the introduced gradient-based approaches in detail, we again first showcase their performance in their default configuration, using the mean of training data as reference point *r* for IG and employing the parameter choices of Kauffmann et al. (2020b) for LRP. The resulting explanations in Table 3 compared to our random noise baselines show that all approaches are capable of finding relevant features. Especially the gradient × input approach shows strong performance on both datasets. While the multiplication with input appears necessary on the ERP data, the raw gradient of the Saliency method reaches comparable performance on the MVTec image data. IG performs well on ERP data but struggles on the MVTec image dataset in its default configuration, and LRP shows low performance on both datasets.

**Table 3:** Mean and standard deviation of gradient XAI performance comparing to ground truth explanations over all anomalies for ERP and MVTec data, respectively.

**XAI**	**ERP**		**MVTec**	
	**ROC**	**COS**	**ROC**	**COS**
Noise	22.7 (7.0)	–28.7 (5.3)	50.2 (1.8)	6.3 (2.7)
Noise × input	52.3 (13.0)	–27.2 (8.2)	32.3 (10.7)	–4.5 (5.5)
Saliency	50.4 (15.9)	6.0 (18.8)	72.4 (5.0)	22.1 (8.0)
Gradient × input	**88.1 (13.0)**	**63.7 (18.5)**	**76.5 (4.4)**	**25.2 (8.7)**
IG	78.8 (14.9)	35.6 (20.7)	64.1 (6.4)	13.5 (7.4)
LRP	65.3 (20.5)	–22.0 (13.9)	65.0 (7.1)	1.8 (3.4)

Best results highlighted in bold.

#### 5.4.2. References *r* for path-based approaches

While the results of the raw gradients in the Saliency and gradient × input methods are in line with observations that the gradient signal does indeed yield explanation properties (Simonyan et al., 2014), many works in the past identified that these explanations are noisy and insensitive to specific signals (e.g., when gradients vanish due to saturated activation functions) (Shrikumar et al., 2016; Sundararajan et al., 2017). One of the proposed solutions, summing gradients along a path to avoid regions where gradients are zero as done in IG, again requires a reference data point as hyperparameter. According to the authors, this reference should be chosen to remove signal (Sundararajan et al., 2017), opening up gradient based approaches to the same issues as perturbation-based approaches with regards to finding a specific reference value that is devoid of anomaly signal, as discussed in Section 4.3.

To show the impact of the choice of reference *r* on path-based explanations, we demonstrate the effect of both established references from image classification such as the mean of training data (mean) and the zero vector (zeros), as well as the anomaly detection specific choices of nearest neighbors (NN) and the optimization scheme in Equation (12) (lopt) which we introduced in Section 4.3. While results in Table 4 show decent performance of IG when using the mean and zeros references from image classification, the nearest neighbor reference performs poorly on the ERP data. The optimization scheme of Takeishi and Kawahara (2020) on the other hand indeed improves performance considerably, yielding very high XAI performance scores on all metrics for both datasets.

**Table 4:** Mean and standard deviation of IG performance on ERP and MVTec data with varying reference point *r*.

***r***	**ERP**		**MVTec**	
	**ROC**	**COS**	**ROC**	**COS**
Mean	78.8 (14.9)	35.6 (20.7)	64.1 (6.4)	13.5 (7.4)
Zeros	84.4 (14.0)	58.2 (20.1)	67.5 (6.3)	17.8 (8.1)
NN	54.9 (13.3)	16.5 (37.4)	70.5 (5.4)	21.0 (9.1)
lopt	**90.8 (11.9)**	**65.8 (21.7)**	**96.2 (2.4)**	**34.5 (10.5)**

Best results highlighted in bold.

**Figure 5:**
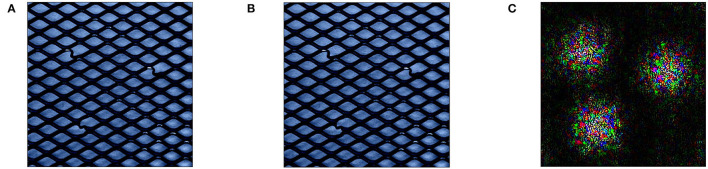
Generating reference *r* through Equation (11) on MVTec: similar to the perturbation issues described in Section 4.3, Equation (11) generates reference points *r* **(B)** that are visually indistinguishable from *x* **(A)**. We again visualize the amplified change in pixel values in **(C)**.

Despite the strong performance, however, an inspection of the created reference points in Figure 5 again shows that this procedure creates adversarial reference points that might skew explanations away from truly meaningful characteristics learned by the model. As seen in Figure 5B, references created through Equation (12) are visually indistinguishable from the original data point in Figure 5A and still retain their anomalous segments (previously highlighted in Figure 2D). While the changed feature values in *r*, which we visualized in an amplified form in Figure 5C shows that changes were indeed made in the vicinity of the three anomalous segments within anomaly *x*, the interpretation of explanations that result from using adversarial reference points *r* that contain normal behavior only for the anomaly detector but not for a human observer is unclear.

#### 5.4.3. Architectural limitations of Layerwise relevance propagation

The alternative approach of LRP avoids the use of reference data points. However, the demonstrated results of Table 3 showed poor performance of LRP compared to other gradient-based approaches. Reasons for this behavior may be found in the architectural limitations of the LRP framework: while Kauffmann et al. (2020b) propose LRP rules that allow it's application on many established differentiable anomaly detection models, the LRP framework is not capable of distributing relevance in scenarios where one layer has multiple input layers. To model common anomaly detection architectures such as autoencoder networks, where the anomaly score is usually extracted from a distance between the input layer and the reconstruction layer of the autoencoder, Kauffmann et al. (2020b) model the input layer as constant in the distance calculation. While this is a necessary assumption to retain the relevance conservation property of LRP, experimental results on the ERP autoencoder show that performance suffers significantly by not assigning a gradient to the input layer, causing LRP to generate considerably lower explanation scores in comparison to other gradient-based approaches in Table 3. Removing this assumption and applying the LRP variant of Kauffmann et al. (2020b) on the ERP autoencoder while retaining the gradient in the distance calculation significantly improves performance as shown in Table 5, but violates the relevance conservation property of LRP. As a result, while LRP successfully avoids the use of a reference data point, it is not readily applicable to common architectural choices such as distance calculations or skip connections. Further research into correctly distributing attribution according to the LRP properties between multiple layers that each possess a gradient with respect to the input is therefore desirable.

**Table 5:** Results of LRP on the ERP autoencoder when keeping the distance layer input constant as in Kauffmann et al. (2020b) and when allowing a gradient flow.

**XAI**	**Variant**	**ROC**	**Cos**
LRP	Constant	65.3 (20.5)	–22.0 (13.9)
LRP	Gradient	88.0 (12.8)	62.2 (19.2)

Performance improves significantly when breaking the conservation property and allowing gradient flow.

## 6. Model-specific explanations

Aside from the previously introduced approaches that operate either entirely model-agnostic or only require a differentiable anomaly detector, multiple works have been proposed to generate feature relevance explanations for specific anomaly detection architectures. In contrast to the previously discussed approaches these methods heavily exploit the structure of the underlying anomaly detector to generate feature relevance explanations.

### 6.1. Depth-based isolation forest feature importance

Carletti et al. (2020) introduce Depth-based Isolation Forest Feature Importance (DIFFI) as an explanation approach for the well-known isolation forest (Liu et al., 2008) algorithm. Isolation forest is an unsupervised algorithm that uses the concept of isolation to identify anomalies using an ensemble of decision trees. The decision trees are generated by randomly splitting the training data until all training points are fully separated. Anomalies are then detected by measuring how fast they arrive on the leaf nodes of the learned trees, noting that points that are quickly isolated at random carry anomalous characteristics that allowed for the isolation. To generate feature relevance scores for single data point decisions made by isolation forests, Carletti et al. (2020) utilize this intuition by traversing a learned tree to the data point and assigning the inverse height of the data point within the tree as relevance to all features that were used as split criteria along the path to the data point. This process is repeated for all trees and feature relevance scores are summed, attributing the isolation of an individual data point to the used splitting features along all paths. Finally, all features are weighted by their inverse occurrence along all paths to counteract an effect on the explanations through the random selection during training of the isolation forest.

Kartha et al. (2021) extend this approach to additionally factor in the imbalance of trees before and after a split criterion, giving more relevance to features that truly isolated the data point to be explained instead of relying purely on the height of the split criterion in the tree.

### 6.2. Principal component analysis-based anomaly detection

Takeishi (2019) presents an approach to extract feature relevance explanations from an anomaly detector based on probabilistic principal component analysis (PCA) (Tipping and Bishop, 1999). This detector learns a linear encoding e:X→Z of data points $X\subseteq {\mathbb{R}}^{d}$ into a latent space $Z\subseteq {\mathbb{R}}^{p}$ with dimensionality *p* < *d* where the data X is decomposed into its eigenvectors and only the *p* dimensions with highest eigenvalues are retained. Points are then reconstructed through an additional linear decoding function d:Z→X and a score of outlierness is obtained through the reconstruction error of applying the transformation through ||*x*−*d*(*e*(*x*))||_2_ for a given data point *x*.

On this linear anomaly detector, Takeishi (2019) obtains feature relevance explanations through Shapley values as described in Section 4.2. While the perturbation approaches of Section 4.2 use reference data *r* to assess the detection output in absence of different features, Takeishi (2019) avoids the use of reference data through calculating the probabilities of removed feature entries directly using the probabilistic component of the anomaly detector.

### 6.3. Neuralization

Kauffmann et al. (2020a) introduce a “neuralization” step for explaining the outputs of one-class support vector machines (OC-SVM) (Schölkopf et al., 2001). In contrast to other model-specific approaches, they do not explain the OC-SVM model directly but introduce a specific transfer procedure, neuralization, that converts a fully trained OC-SVM into a neural network representation, allowing the subsequent application of gradient-based explanation approaches such as the works discussed in Section 5. Their proposed procedure transfers the final outlier scoring function learned by the OC-SVM to a two-layer neural network that mimicks the behavior of the OC-SVM. Through this conversion they are able to apply an LRP-style XAI approach as introduced in Section 5.3 to generate feature relevance explanations. The authors further apply this “neuralization” approach to the anomaly detection approach of kernel density estimation (Rosenblatt, 1956) in subsequent work (Kauffmann et al., 2020b).

### 6.4. Limitations

The development of highly model-specific XAI approaches bears significant potential in multiple areas. While the close connection to the model architecture might allow for improved computational efficiency over model-agnostic approaches (Carletti et al., 2020), the exploitation of model characteristics is also a promising way to circumvent current issues of feature relevance XAI approaches such as the choice of reference data as demonstrated by Takeishi (2019) on PCA. Finally, mapping fully trained anomaly detection models to alternative representations as done by Kauffmann et al. (2020a) is a promising procedure that allows the re-use of XAI approaches that have been identified as reliable in the domain.

While the continuous development of model-specific explanations approaches can therefore provide numerous benefits to the domain of feature relevance XAI in anomaly detection, the main limitation of this type of approach is the restriction to the specific anomaly detection model. In areas where explainability is considered as a requirement of anomaly detectors, this may limit the performance of available detectors in cases where a model-specific explanation framework is not available for the best performing anomaly detection architecture. Especially on ERP data, hyperparameter studies of Tritscher et al. (2022b) show isolation forests and PCA-based anomaly detection to perform considerable worse than other architectures, which limits the application of model-specific XAI approaches such as DIFFI or the Takeishi (2019) method for explaining anomaly detection of PCA. Beyond potential limitations of anomaly detection performance, the promising procedure of Kauffmann et al. (2020a), who map anomaly detectors to different architectures, also comes with the limitations of the XAI approach that is applied after the mapping, requiring not only the mapping itself but also an XAI approach that is capable of producing reliable explanations on the resulting mapped architecture.

## 7. Discussion

In this work, we reviewed XAI approaches that explain single decisions of anomaly detectors by highlighting which features are most anomalous. We systematically structured these feature relevance XAI approaches by their access to training data and anomaly detector. We introduced the feature relevance approaches and their existing adaptations to anomaly detection in detail, and showcased their current limitations.

We showed that the many highly performing XAI approaches employed in anomaly detection require the manual selection of a reference data point. This proves problematic in anomaly detection as commonly used choices for reference data from other domains such as classification do not transfer to anomaly detection.

One approach that addresses this problem by finding optimal reference data through optimization considerably improves XAI performance in our showcase, but suffers from generating adversarial data points that fall outside the training data manifold. As this issue is commonly investigated within the research area of counterfactual explanations (Guidotti, 2022), incorporating techniques to avoid these adversarial data points during optimization constitutes a promising area for future work.

As another approach to circumvent issues that arise from reference data points in anomaly detection, we discussed model-specific XAIs that use the model architecture to avoid the use of reference data entirely. While this is a promising solution to avoid common issues with reference data, this area of research requires specific design decisions for individual anomaly detectors. Therefore, developing model-specific XAI approaches to ensure that state-of-the-art architectures can be explained without the use of reference data is an interesting research direction.

Finally, once reliable XAI approaches are found within the anomaly detection domain, the extension of conversion procedures that transfer trained anomaly detectors such as one-class support vector machines or kernel density estimation to a more easily interpretable framework becomes a promising research area that allows the transfer of reliable XAI approaches to state-of-the-art architectures.

## Author contributions

JT designed the showcases and wrote the paper. AK and AH critically revised the paper and experiments to meet high research standards. All authors contributed to the article and approved the submitted version.

## Footnotes

^1^Code for our showcases is available at https://professor-x.de/feature-relevance-AD.
